# Is there any way to increase consumers’ purchase intention regarding surplus food blind-boxes? An exploratory study

**DOI:** 10.1186/s40359-024-01587-y

**Published:** 2024-02-28

**Authors:** Jie Sun, Yanan Wang, Chun Yang, Jiangjie Chen, Wei Wei, Wei Miao, Hanchu Sun, Chao Gu

**Affiliations:** 1 College of Arts and Design, Zhejiang A&F University, Hangzhou, 311300 China; 2 Xia Qing Communication School, Handan University, Handan, 056005 China; 3 School of Design, Jiangnan University, Wuxi, 214122 China; 4 School of Textile Garment and Design, Changshu institute of technology, Changshu, 215500 China; 5 The Faculty of Industrial Design Engineering, Delft University of Technology, Delft, 2628 CE The Netherlands; 6 Academy of Arts & Design, Tsinghua University, Beijing, 100084 China

**Keywords:** Surplus food, Blind box, Purchase intention, Continuous intention, Recommendation intention

## Abstract

As an emerging business model, merchants are selling surplus food in blind boxes, which provides new opportunities for reducing waste and promoting sustainable development. Surplus food blind boxes, however, have not been extensively researched in terms of design and marketing strategies. Therefore, we are examining how to increase consumers’ purchase intention regarding surplus food blind boxes as well as the relationship between purchase intention, continuous intention, and recommendation intention. In this paper, two studies are conducted. As part of study 1, We used factor analysis to explore the reasons that influence consumers to purchase. In study 2, we utilized structural equation modeling to examine the impact of purchase intention on continuous intention and recommendation intention. Results indicate that food quality, perceived sustainability, and conspicuous consumption are the most important positive factors. The most important negative factors are perceived food risk, resistance to sales techniques, taste anxiety, and inadequate marketing strategies. Based on our findings, we constructed a path relationship between purchase intention, continuous intention, and recommendation intention. The purchase intention of the surplus food blind box users directly influences the recommendation intention and the continuous intention, while the recommendation intention indirectly influences the continuous intention. This study did not demonstrate that gender had a moderating effect. Meanwhile, it provides a reference for the actual marketing management of surplus food blind box merchants.

**Supplementary Information:**

The online version contains supplementary material available at 10.1186/s40359-024-01587-y.

## Introduction

### Research background

A global problem, food waste is a reflection of issues related to the production and consumption of food. In accordance with statistics published by the Food and Agriculture Organization of the United Nations (FAO), about one-third of food produced for human consumption worldwide is discarded or wasted. It is estimated that by 2030 annual food waste will increase by a third, equivalent to 66 tons of food wasted every second, according to the Boston Consulting Group (BCG) [1]. The waste of food not only causes a waste of resources, but also poses an ecological threat. Approximately 26% of global greenhouse gas emissions are attributed to the food industry, of which 6% are attributed to edible food that is yet to be consumed [2]. The methane released from the decomposition of food waste in landfills contributes 25 times more to global warming than carbon dioxide [3]. Consequently, developing ways to effectively utilize this part of edible food that is at risk of being discarded, minimizing food waste, and reducing the need for scarce resources like land and water to handle food waste will assist in the development of a sustainable food consumption system.

Unsustainable food systems are largely attributed to food waste [4]. The concept of food waste refers to the discarding of food that is suitable for consumption in the food supply chain or the creation of expired foods as a result of poor inventory management and economic behavior [5]. Several studies have attempted to address the problem of food waste through various means: public awareness campaigns [6]; coordinating communication between retailers and agricultural producers [7]; improving management [3]; improving food packaging [8]; applying new technologies [9]. Currently, research is being conducted in an effort to reduce food waste by raising awareness among social groups, creating new integrated supply chains for producers, and improving the aesthetics of products. Presently, some food and catering businesses are experimenting with a new food sales model called surplus food blind boxes.

Food merchants sell surplus food in blind boxes at a low price, which can reduce losses resulting from unsalable surplus food and at the same time benefit consumers. Food surpluses can be reused by redistribution and human consumption [10]. By maximizing the value of surplus food, we will be able to reduce food waste. It has been estimated that 250 million pounds of food waste have been reduced in Europe and the United States as a result of the implementation of surplus food blind boxes [10]. Most food waste occurs during the distribution and consumption of food [11]. Food manufacturers may have difficulty in accurately identifying the uncertain and heterogeneous needs of consumers when exploring new markets and promoting new products. Surplus food blind boxes provide merchants with a way to develop and promote new products to some extent. A blind box sale is a complementary method to traditional sales tactics that prevents price discrimination by heterogeneous consumers, reduces the mismatch between uncertain demand and production capacity, and helps merchants promote their products [12]. Food blind boxes with superior cost performance may attract new consumer groups for businesses. A blind box may solve the problem of food waste, but it may also improve brand communication by bringing consumers a positive consumption experience. In addition, surplus food blind boxes appeal to young consumers’ curiosity-seeking and gambling psychology [13], so they can stimulate their purchasing behavior to a certain extent. As well as providing a solution to people who have difficulty choosing takeaway meals [10].

Originally, blind boxes were small toys distributed through vending machines, because they were sealed in the box and were not visible, so they were a source of surprise and suspense for consumers. By offering blind box sales, merchants have the opportunity to match supply with demand, resulting in a more efficient inventory clearance process [12]. Chinese blind box companies represented by POP MART have introduced exquisitely packaged blind box toys, which are popular with young consumers [14]. As a result of blind box sales, where the exact product is not known before opening the package, a new buying trend has developed among Chinese consumers [15]. Tmall’s post-95s purchase list shows that blind box sales are growing at a rapid pace in China [16]. To Good to Go in Denmark introduced the surplus food blind boxes in 2015. Users can select their favorite food sellers online to buy the surplus food blind boxes for about 4 euros, and pick them up at their door based on the scheduled pick-up time [10]. Surplus food blind boxes consist of edible foods that were not sold by the merchant on that particular day, and consumers are attracted to them as they are an uncertain consumption model [16]. In this way, edible food waste can be reduced to a great extent [10]. China is currently based on WeChat mini-programs as the main sales channels for surplus food blind boxes, such as Xishi Magic Bag, Rice Blind Box, Pocket Package, etc., as the main sales channels for surplus food blind boxes. It has been operating in Shanghai, Changsha, Chengdu, Wuhan, Hangzhou, Hefei and other Chinese cities for a number of years [17]. Despite the fact that surplus food blind boxes have already been used by a certain number of people, attitudes towards them are still mixed [18].

UN Sustainable Development Goals (SDGs) propose “reducing food losses in production and supply chains” as a solution to food waste [19]. From agricultural production to households and restaurants, food for consumption is a component of food chin [20]. As a new method of food consumption in line with circular food ecology, surplus food blind boxes help food manufacturers reduce excess inventory and provide a way to match supply and demand for food consumed in this area of the food chain. Using surplus food blind boxes as a low-cost means of reducing edible food waste in the supply chain, to involve as many participants as possible, may prove to be an effective way of reducing food waste. As the world’s most populous and largest developing country, China is also challenged with the issue of food waste. In China, it is estimated that each person wastes approximately 93 g of food per meal, which translates into about 279 g per day [21]. Restaurant food waste is also increasing because of urbanization and the expansion of the catering industry in China. Approximately 55.86% of China’s urban domestic waste comes from the kitchen [22]. The emergence of surplus food blind boxes has allowed catering businesses to once again sell leftover edible ingredients [17, 18]. In some ways, surplus food blind boxes help to reduce food waste. It is, however, necessary to develop sustainable marketing methods that meet the needs of consumers in order to reduce the waste of surplus edible food [23]. Initially, consumers may purchase surplus food blind boxes due to the novelty of the concept. How to make consumers form a continuous purchase intention will determine whether surplus food blind boxes can reduce edible food waste over time. At present, the research on the surplus food blind box focuses on how to use the surplus food blind box to improve consumers’ purchase intention [10]. Therefore, this study aims to analyze the positive and negative factors that influence consumers’ purchase intentions to surplus food blind boxes, and establish a path relationship between purchase intention, recommendation intention, and continuous intention, as well as a moderating effect of gender. To provide information on the design of surplus food blind boxes and to develop a theoretical framework for the sustainable marketing of surplus food blind boxes.

## Theoretical framework and research hypotheses

### Theoretical framework development attention-interest-search-action-share model (AISAS)

**Fig. 1:**
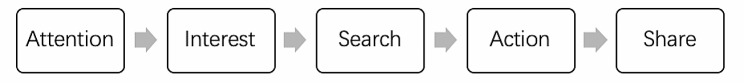
AISAS model

With the popularity of mobile devices and the change in the way consumers receive Internet messages from passive to active sources, Dentsu proposed AISAS to describe consumer behavior more accurately [24]. When consumers actively search for products with certain functions on the Internet, they may be triggered to make a purchasing decision as opposed to passive advertisements on traditional media such as television, newspapers, and magazines [24]. Social networks illustrate this model nicely. When a user notices a product or advertisement, he or she may be interested in actively seeking relevant information and develop that into a purchase action. Following use, the user will share their experience on the Internet, thus completing a closed loop. A consumer’s active search for feedback from other consumers may seems to be more persuasive and objective than official brand advertising [25, 26]. AISAS is used to analyze the marketing communication methods used by vloggers on social media platforms [27]. Additionally, research has been conducted on the use of the AISAS model to modify the marketing evaluation model for e-commerce platforms [28]. In light of AISAS, we propose to investigate the consumer’s PI of surplus food blind boxes, which may contribute to developing positive consumption sharing, thereby increasing the size of the market. The AISAS model is illustrated in Fig. 1.

### Purchase intention (PI)

An individual’s purchase intention (PI) is the degree to which he or she intends to purchase a particular product or service, or a particular brand of products and services [29]. Individuals with stronger PI for food are more likely to purchase such items effectively [30]. According to this study, it refers to a consumer’s intention to purchase blind boxes of surplus food. Through the prediction of PI, it is possible to more effectively predict consumer behavior [31]. By studying the factors that influence the PI of surplus food blind boxes by consumers, it may be possible to predict the PI and the purchasing behavior of surplus food blind boxes more accurately. Therefore, it is essential to examine what factors have a positive and negative impact on consumers’ PI, as well as their relationship with other factors, in order to promote the sustainable marketing of surplus food blind boxes. In recent years, marketing has increasingly focused on the positive impact of uncertainty [16, 32]. It remains to be determined whether the uncertainty created by blind boxes of surplus food is also beneficial for the marketing of edible leftover food. By 2020 young Chinese consumers will dominate consumption, and they prefer products that are unique and exciting to buy [16]. Since strong intentions are associated with strong behaviors, it may be possible to infer the probability of users purchasing surplus food blind boxes in the future based on PI [33]. It is necessary to further investigate the impact of PI on the marketing process of surplus food blind boxes.

### Continuous intention (CI)

Continuous intention (CI) is the behavior that consumers adopt after using the surplus food blind boxes [34]. The sustainability of marketing the surplus food blind boxes depends on users continuing to use the service rather than first deciding to make use of the service [35, 36]. In order to reduce the waste of edible food, the sustainable marketing of surplus food blind boxes is essential; therefore, it is imperative to find a way to improve the CI of consumers of surplus food blind boxes. As part of the product evaluation process, one method of measuring the success of the product is the CI that is formed by the user for the blind box of surplus food [37, 38]. Blind boxes containing surplus food are different from blind boxes containing dolls, and blind boxes containing leftover fresh ingredients are also different from regular food blind boxes. It is uncertain what the remaining ingredients will be in the surplus food blind box, which are based on what the merchant has actively prepared on that day. In marketing, uncertainty has been shown to have a positive effect [16, 32]. To some extent, the uncertainty of a blind box of surplus food appeals to the gambling psychology of some consumers [39]. It is still undetermined whether the user will continue to purchase the surplus food blind boxes after their sense of novelty fades. What factors may affect the purchase of surplus food blind boxes, still needs to be determined. We have found that there is still some gap between users who have PI and those who implement purchasing behavior in the area of sustainable clothing, and in order to promote sustainable purchasing behavior, it is necessary to take proactive measures [40]. A further study is needed to determine whether consumers’ PI can positively influence their CI to purchase surplus food blind boxes. In addition, in research on consumers’ perceptions of brands, gender plays a moderating role in the impact of brand perceptions on PI. In comparison with men, women are more likely to perceive warmth in information, and perceived warmth has a moderate effect on PI [41]. Moreover, gender was suggested to have a moderating effect on the use of interaction to motivate consumer interaction in the social e-commerce industry [42]. We intend to investigate whether the gender has a moderating effect on the path relationship between PI and CI of surplus food blind box. Therefore, this study hypothesizes the following:

#### H1a

The PI of the user who purchases the surplus food blind boxes has a positive impact on the CI.

#### H1b

Gender exerts its moderating impact on the relationship between PI and CI.

### Recommendation intention (RI)

Recommendation intention (RI) is the psychological behavior of recommending products to family members, colleagues, and friends [43]. According to this study, RI is defined as consumers’ willingness to perform recommendation behaviors after using the surplus food blind boxes through various channels, such as verbal recommendations and social media sharing etc. In the study of digital influencers and followers, it has been found that the influencer’s recommendation leads to good word-of-mouth for the brand, and followers have an intention to purchase the product, which is considered to be a significant factor in PI [44]. Additionally, it is a key indicator of service quality [45]. Therefore, the role of RI in the blind box marketing of surplus food needs to be clarified. Studies have shown that when users enjoy using a product or service, they are more likely to recommend the product or service to others [46]. Consumers may have PI because of the uncertainty associated with surplus food blind box products, however after purchasing it, different consumers will experience different feelings such as surprise and disappointment. There are some consumers who may feel that purchasing surplus food blind box products that they have no control over is a risky activity. According to Chen, the risks that users may face will negatively affect the RI [46]. Therefore, it remains to be determined whether the PI of surplus food blind box users will positively affect RI. According to research, referrals are several times more effective than advertising [46, 47]. Increasingly more channels of recommendation are being developed through mobile Internet and social media, and the audience is expanding. It has not been widely popularized yet to use blind boxes for surplus food. There is a possibility that RI may play a significant role in the promotion and sales of surplus food blind boxes [48]. According to behavioral research, RI is an important indicator of user loyalty [45, 49]. Consumers who have a high RI are likely to be more loyal to surplus food blind box products and to be able to continue purchasing it. Further research is needed to determine whether RI and CI of surplus food blind box consumers are positively correlated. Based on the demographic characteristics of hala, it has been found that men are more satisfied with hala food than women, so men are more likely to recommend hala food to friends and family [50]. This study will examine the relationship between gender and RI, PI, and CI in the surplus food blind box study. The following assumptions are made based on the above theories:

#### H2a

The PI of the user who purchases the surplus food blind boxes has a positive impact on the RI.

#### H2b

Gender exerts its moderating impact on the relationship between PI and RI.

#### H3a

The RI of the user who purchases the surplus food blind boxes has a positive impact on the CI.

#### H3b

Gender exerts its moderating impact on the relationship between RI and CI.

### Research purpose

This research consists of two studies designed to increase acceptance of surplus food blind boxes. Study 1 examines the subjective reasons that influenced consumers to choose surplus food blind boxes, analyzes the reasons that may have positive and negative effects on consumers’ purchases of surplus food blind boxes. Study 2 established a path relationship structure consisting of consumers’ PI, CI and RI of surplus food blind boxes, as well as the moderating effect of gender. Based on the results of Study 1 and Study 2, we hope to understand the factors that influence PI from the perspective of consumers and evaluate the impact relationship between variables in order to provide merchants with reference strategies to support blind box sales of surplus food.

## Research method

**Fig. 2:**
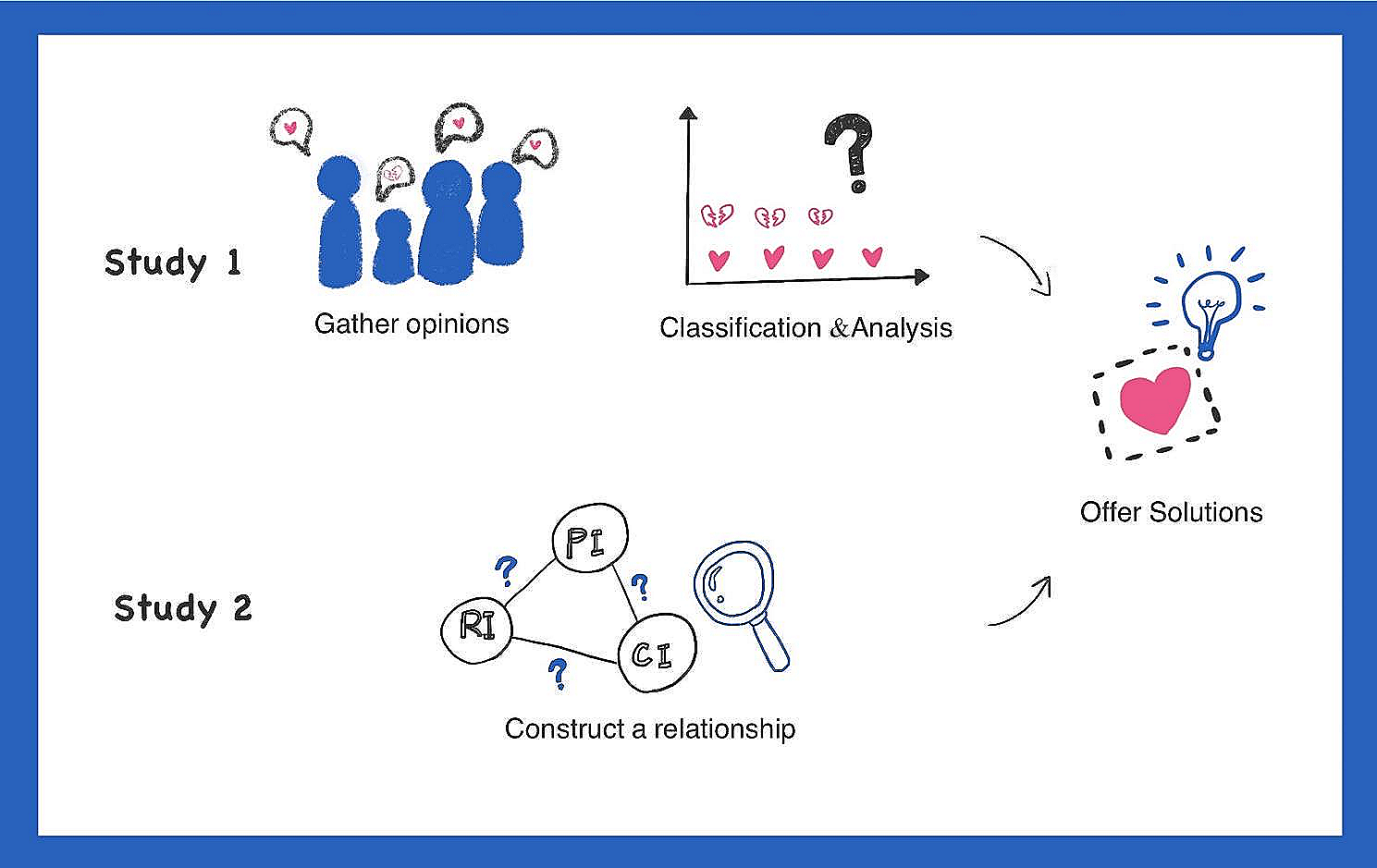
Research design workflow

The study consists of two parts, study 1 and study 2. The purpose of Study 1 is to develop an inductive analysis of the positive and negative factors that affect consumers’ PI. Study 2 develops a theoretical model of consumer behavior when choosing surplus food blind boxes. In Study 1, we identified reasons that may motivate consumers’ PI of surplus food blind boxes. However, for a broader understanding of user behavior and consumer motivations, users’ CI and RI are also worthy of consideration, which is why we design study 2 to highlight their importance in marketing. Figure 2 illustrates the research process.

### Study 1- factors influencing consumers’ PI of surplus food blind boxes

#### Study design

In Study 1, we examine the specific factors that have positive and negative impacts on consumers’ choice of surplus food blind boxes in the actual consumption process. Based on an analysis of surplus food blind boxes, we propose sustainable marketing solutions and assist merchants in better understanding user needs and improving marketing strategies. Two rounds of questionnaire surveys were conducted to investigate the factors affecting consumers’ PI of surplus food blind boxes. During the first round of questionnaires, users were asked to answer an open-ended questionnaire, as shown in the appendix, which sought to determine the three reasons that influence their PI of surplus food blind boxes.

Following this, the results of the first round of questionnaires were classified and sorted, as shown in the appendix. The second round of questionnaires was formulated based on the results of the first round. Using SPSS software, the results of the second round of questionnaire surveys were analyzed in order to summarize the factors that influence consumers to purchase surplus food blind boxes.

#### Respondents

In order to conduct this study, we commissioned an authoritative market research company Wenjuan Xing. We asked the company to identify subjects with experience in buying surplus food blind boxes and to try to select subjects with gender, age, monthly income, educational background, occupation, and regional characteristics that are representative of the general population in mainland China. Except for the age part, are based on the main age distribution of the subjects chosen in previous studies [10]. The advantage of this approach is that the characteristics of the sample are close to those of the population we wish to study, making us more likely to reach consumers who may be exposed to or purchased such goods in actual management, and providing valuable suggestions for operators.

#### Sample size

We developed our sample size based on survey scales used in previous studies involving quantitative research methods in order to improve the accuracy of our research. According to Marsh, Balla and McDonald (1988), a sample size of at least 200 is required if *r* = 3 [51]. There are two surveys in Study 1. In the first round of survey, resulted in 318 questionnaires with 954 responses. The reasons that impact PI positively have 29 meaningless responses and 925 valid responses. The reasons that impact PI negatively have 63 meaningless responses and 891 valid responses. In this instance, it is necessary to clarify that some people pay more attention to one question. For another question, they may think that something will never happen, or they cannot comprehend the situation, or they lack patience, which results in responses such as “Don’t know”, “/“, “I don’t think I will buy/won’t buy”. We mark similar specific responses in the questionnaire as meaningless responses, rather than treating the entire questionnaire as invalid. The second round of the survey involved 480 respondents. After eliminating invalid questionnaires, 432 valid questionnaires were collected, resulting in an effective rate of 90%.

#### Sampling technique

We used stratified random sampling in this study. In the stratification, the subjects are considered to meet the following characteristics of mainland Chinese individuals. A relatively balanced ratio of males to females; Due to the fact that China is a developing country with a large population. In general, there are very few people with average salaries that exceed 18,001, below 4000, 4001–6000, 12,001–18,000 are average, and most people have an average salary within 6001–12,000; Due to the spread of compulsory nine-year education in China and the rapid pace at which universities are being constructed, the country is actively promoting young people to have more opportunities to pursue higher education. Therefore, there are very few junior high school or below and the number of people who have completed high school or secondary school and institute including above is average, while the number of people who are enrolled in undergraduate or college accounts for the majority; Although China has a large farmer population, since surplus food blind boxes are marketed primarily in cities, we did not include the farmer group in our study. Since China has experienced an increase in urbanization, the number of clerks has risen more than any other group, accounting for the majority of the occupation. Additionally, we refer to previous studies’ age distribution.

#### Research instrument

In the first round, we explore two open questions about the positive and negative effects of consumers’ PI of surplus food blind boxes. Subjects were instructed to summarize three reasons for each question. As a result, 25 positive reasons and 25 negative reasons were identified. In the second round, these reasons were used as questionnaire items. Using the analysis of reasons in the first round, the second round was conducted, and each item was rated on a 5-point Likert scale (1 strongly disagrees, 5 strongly agrees). Additionally, two reverse questions are designed to eliminate inconsistent and illogical scales. Appendix provide details of the questionnaire.

#### Data collection

The first round of surveys was conducted from the perspective of consumers. A questionnaire survey was conducted between November 14 and November 20, 2022. In the first round of the questionnaire survey, the reasons influence consumers’ PI toward surplus food blind boxes were surveyed. From November 29 to December 5, 2022, a second round of surveys was conducted. Online questionnaires are still used as a method of conducting surveys. The participant has the option to opt out of the survey at any time. When they have completed the questionnaire and submitted it, they will be able to receive payment online. Participants completed the questionnaire voluntarily and under the principle of consent.

#### Data analysis

In the first round of survey, we gathered and sorted all the reasons mentioned in open questionnaires that influenced consumers’ PI of surplus food blind boxes in positive and negative ways. As an example, we collected information on the reasons that influence PI, and there were various expressions of similar concepts such as: cheap, affordable, could save money, discounts, etc. Therefore, in this step of the conceptual arrangement, they are combined into cost effectiveness. Order does not matter since every question will be asked and each has an independent meaning. In accordance with this method, we combined responses with similar meanings, and after removing meaningless responses, we extracted 25 factors for both positive and negative responses. In the second round of survey, based on the 50 new factors identified in the first round of the survey, factors with higher loading factors were eliminated. These cross-loading factors had higher loading factors, but there was little difference between the new factors and the old factors. Having tried multiple rotation methods, we decided to remove this part of the loading factors as well as those factors with commonality less than 0.4, factor loadings less than 0.5, and factor loadings with differences less than 0.1. By removing the cross-loading factor, remaining 17 positive factors and 21 negative factors, as shown in Tables 2 and 3. We process employs exploratory factor analysis, chooses a maximum variation method, and used principal component analysis to extract new factors with eigenvalues greater than 1. Positive reasons included Kaiser-Meyer-Olkin value of 0.926 as well as Bartlett’s Sphericity Test’s Chi-Square of 3138.174 with a significance level of 0.000. In terms of negative reasons, Kaiser-Meyer-Olkin value = 0.929. The Chi-Square of Bartlett’s Test of Sphericity was 3638.995, Sig is 0.000. According to the results of the two exploratory factor analyses, KMO values were greater than 0.05, and Bartlett’s Test of Sphericity was significant (*p* <.05), indicating that factor analysis could be undertaken [52]. In an analysis of factors that positively influence consumers to purchase surplus food blind boxes, 3 new factors with Eigenvalues greater than one have been identified. The total variance explained was 57.709%. In an analysis of factors that negatively influence consumers to purchase surplus food blind boxes, 4 new factors with Eigenvalues greater than one have been identified. The total variance explained was 57.476%. It was found that the communalities of positive and negative factor were greater than 0.4, and factor loading was greater than 0.5, in the analysis of both positive and negative factors. Therefore, additional factors did not need to be removed [53]. We named the newly extracted factors based on the main content to which most of the items refer [54, 55]. As an example, the new factor conspicuous consumption consists of fashionable, love the design of packaging, recommendation and being superior.

### Study 2- verify the relationship between PI, RI, and CI of surplus food blind boxes purchase

#### Study design

Surplus food blind boxes provide a means of reducing edible food waste and promoting the development of low-carbon and recyclable foods. In study 2, the role of PI with CI and RI in marketing promotion was examined. In addition, verify whether gender has a moderating effect. The questionnaire is detailed in Table 5.

#### Respondents

In study 2, we still employed the services of an authoritative market research firm Wenjuan Xing to conduct the research, and the subjects had to have experience in purchasing surplus food blind boxes, while at the same time, each characteristic was in agreement with the distribution of the general population on mainland China. For the age distribution of the sample, we consulted previous studies [10].

#### Sample size

A total of 750 responses were collected in this study, of which 569 valid questionnaires were recovered. According to Hair, Gabriel [51], this sample size is appropriate for the construction of structural equation models. 569 valid questionnaires, and the ratio (p:n) of the estimated parameter to the sample that meets Jackson’s maximum likelihood method is greater than 1:10 [59].

#### Sampling technique

Study 2 samples are drawn from the same parent population as study 1. Apart from the age reference from previous studies, the remaining samples are representative of mainland Chinese residents and have been collected using the same sampling method. Based on the consistency of sampling techniques and respondents, it can be concluded that the phases of this study are coherent, and that the respondents are homogeneous in nature.

#### Research instrument

This study contains 20 questionnaire items, 7 of which are basic information about the subjects. In measuring the factors that influence consumers’ PI of surplus food blind boxes, we used a five-level Likert scale. The items were developed based on existing instruments that have been validated in relevant literature. The subject of all questionnaires was adjusted to surplus food blind boxes without changing the meaning of the question. As part of this study, the 4 items proposed by Wang, Pacho [56] were used to measure the user’s PI; the 4 items proposed by Zanetta, Hakim [57] were used to measure the user’s CI; and the 4 items proposed by Al-Ansi et al. 3 items and 2 items proposed by Correia et al. were used to measure the user’s RI [50, 58]. In addition, we set up two reverse questions in order to eliminate subjects with inconsistent logic.

#### Data collection

As in study 1, we collected data through an online survey, which was distributed in October 2022. The subjects participated in the survey voluntarily and were free to withdraw at any time. They will be paid after completing the questionnaire.

#### Data analysis

In this study, 750 samples were collected. The questionnaire contains logically opposite questions. If the subject’s answers are logically inconsistent, the questionnaire will be deemed invalid and discarded. Similarly, if all subjects choose the same answer when answering the questions, there is a high likelihood that they did not read and fill in the questions carefully, and the questionnaire will be considered invalid and discarded. A total of 569 valid samples were recovered after excluding invalid samples with logical problems and too many identical options in accordance with this principle, resulting in an effective rate of 75.87%. Firstly, we used SPSS to analyze the reliability of the data, and Cronbach’s Alpha and CITC to test the scale’s internal consistency. Secondly, AMOS was used to conduct a confirmatory factor analysis (CFA) on the data. Thirdly, in order to analyze the discriminant validity of our data, we used two methods: Fornell-Larcker criterion and heterotrait-monotrait ratio (HTMT). Finally, we utilize AMOS to construct a structural equation model to analyze the relationship between PI, CI, and RI of users purchasing surplus food blind boxes. The path coefficients of male and female subjects in the same model can be compared to determine whether gender has a moderating effect on PI, CI, and RI.

## Results

### Study 1- factors influencing consumers’ PI of surplus food blind boxes

In this study, the causes of positive and negative impacts on surplus food blind boxes PI were examined. Table 1 shows the distribution of various demographic variables in two surveys of study 1.

**Table 1:** Demographic characteristics of the respondents of survey 1 & 2

Sample	Category	Survey 1		Survey 2	
		Number	Percentage (%)	Number	Percentage (%)
Gender	Male	156	49.057	182	42.130
	Female	162	50.943	250	57.870
Age	Under 18	1	0.314	5	1.157
	19–29	147	46.226	182	42.130
	30–39	141	44.340	208	48.148
	40–49	18	5.660	29	6.713
	Over 50	11	3.459	8	1.852
Marital status	Married	94	29.560	321	74.306
	Unmarried	224	70.440	111	25.694
Monthly Income (RMB)	Below 4000	46	14.465	49	11.343
	4001–6000	63	19.811	94	21.759
	6001–12,000	147	46.226	194	44.907
	12,001–18,000	45	14.151	72	16.667
	18,001 or more	17	5.346	23	5.324
Education	Junior high school or below	1	0.314	1	0.231
	High school or secondary school	10	3.145	15	3.472
	Undergraduate or college	276	86.792	386	89.352
	Institute including above	31	9.748	30	6.944
Occupation	Civil servant	27	8.491	40	9.259
	Clerk	153	48.113	210	48.611
	Worker	53	16.667	69	15.972
	Public service agency	21	6.604	36	8.333
	Student	31	9.748	33	7.639
	Self-employed	33	10.377	44	10.185
Area	Eastern china	191	60.063	266	61.574
	Central china	69	21.698	77	17.824
	Western china	32	10.063	67	15.509
	Northeast china	25	7.862	21	4.861
	Hong Kong, Macao and Taiwan in China	1	0.314	1	0.231

Based on the results of the first round of open-ended questionnaires, we have identified 25 reasons for positive or negative impacts on consumers’ purchase of surplus food blind boxes. By removing higher cross-loading factor, the remaining 17 positive factors (as shown in Table 2) and 21 negative factors (as shown in Table 3) were re-analyzed. Detailed extraction results were presented in Table 2 for factors that positively influence the purchase of consumer surplus food blind boxes, while Table 3 shows the extraction results for factors that negatively influence the purchase of consumer surplus food blind boxes.

As shown in Table 2, there were several factors that positively influence consumers’ decisions to purchase surplus food blind boxes. In total, 7 items comprised the first new factor food quality. It contained information regarding the quality of the flavor, nutritional combination, sanitation and safety, and storage methods of the surplus food blind boxes. A total of 6 items comprised the second new factor perceived sustainability. Relevant topics include cost-effective pricing of surplus food blind boxes, novelty and fun, and the protection and recycling of resources. In order to preserve remaining edible food, surplus food blind boxes provide a new solution. The economic and environmental benefits of surplus food blind boxes were positive influences on consumers’ purchasing decisions. We have named the third new factor conspicuous consumption in recognition of the popularity of consuming surplus food blind boxes. There were 4 items that constitute the third extracted factor: “feel fashion”, “like packaging design”, “recommended by others”, and “superiority”. As a point of clarification, superiority means that in the Chinese context of the respondent, one can show that one has superior taste, philosophy, or values when purchasing blind boxes of surplus food. It was less important to consider the functional properties of the surplus food blind boxes themselves, but rather what the surplus food blind boxes represent to the outside world when choosing to consume them.

Table 3 illustrated the analysis of negative influences on the purchase of surplus food blind boxes by consumers. The first new factor extracted from the data was named perceived food risk. A total of 5 items comprised the first new factor. As with the first new factor extracted from the positive impact, it was related to the food itself, but here consumers are concerned about the unknown risks associated with the surplus foods in blind boxes. A consumer’s choice of surplus food blind boxes was driven by their concerns regarding safety, hygiene, health, and food quality of the remaining ingredients. The second new factor was resistance to sales techniques. According to the second new factor, a total of 7 items were removed primarily since surplus food blind boxes were not accepted or liked psychologically. The purchase of surplus food by some consumers was considered unethical, and they think that there was no need to purchase surplus food blind boxes. The third new factor was named taste anxiety. 5 items comprised the 3rd new factor, which included worries about buying food that did not meet personal tastes, and psychological gaps caused by disappointing expectations. The fourth new factor was inadequate marketing strategies. There were 4 items comprised the fourth new factor, namely “restricted purchase channels”, “complicated purchase methods”, “worry about insufficient food in blind boxes”, and “worry about too single food in blind boxes”. The factors such as purchasing channels, methods, and product designs were primarily due to problems in the product marketing process, which prevent consumers from choosing to consume surplus food blind boxes.

**Table 2:** Factors contributing to the positive aspects of purchasing surplus food blind boxes

Coding	Factors	Communalities	Factor loading	New factors	Eigenvalue	Total variance explained	New factors name
J1	Tastes good	0.551	0.666	1	6.931	40.771%	food quality
J2	High quality	0.564	0.712	1			
J3	Reasonable mixture of ingredients	0.513	0.557	1			
J4	Clean	0.699	0.801	1			
J5	Easy to store food	0.573	0.702	1			
J6	Security	0.707	0.811	1			
J7	Nutritional balance	0.537	0.618	1			
J8	Discounted price	0.456	0.640	2	1.728	50.934%	perceived sustainability
J9	Interesting	0.470	0.576	2			
J10	Reduces waste	0.644	0.766	2			
J11	Eco-friendly	0.634	0.705	2			
J12	Suitable for developing a saving habit	0.502	0.605	2			
J13	Make use of resources	0.590	0.691	2			
J14	Fashionable	0.600	0.714	3	1.152	57.709%	conspicuous consumption
J15	Love the design of packaging	0.616	0.759	3			
J16	Recommendation	0.523	0.635	3			
J17	Being superior	0.632	0.745	3			

**Table 3:** Factors contributing to the negative aspects of purchasing surplus food blind boxes

Coding	Factors	Communalities	Factor loading	New factors	Eigenvalue	Total variance explained	New factors name
X1	Not fresh, expired	0.590	0.699	1	7.486	35.649%	perceived food risk
X2	A lack of after-sales service	0.714	0.803	1			
X3	Hygiene problems	0.676	0.801	1			
X4	Concerned about food safety	0.701	0.806	1			
X5	Concerned about the health consequences	0.529	0.642	1			
X6	Unacceptable psychologically	0.613	0.660	2	2.213	46.187%	resistance to sales techniques
X7	Do not like this format	0.634	0.706	2			
X8	Not necessary	0.577	0.709	2			
X9	Someone else may have eaten it	0.568	0.593	2			
X10	Feel that the buying process is tedious	0.651	0.738	2			
X11	Fear of losing face in front of others	0.518	0.600	2			
X12	Don’t like the design of the package	0.524	0.623	2			
X13	Be concerned about buying dislike food	0.533	0.604	3	1.362	52.673%	taste anxiety
X14	Tasteless	0.559	0.526	3			
X15	Anxiety about being cheated	0.509	0.524	3			
X16	An inability to control the unknown	0.579	0.713	3			
X17	Concerned about psychological gaps	0.457	0.501	3			
X18	There are limited channels of purchase	0.510	0.661	4	1.009	57.476%	inadequate marketing strategies
X19	A complicated purchasing process	0.578	0.705	4			
X20	Concerned about a lack of food in the blind box	0.504	0.647	4			
X21	Concerned about the lack of variety in blind box food	0.546	0.567	4			

### Study 2- relationship between PI, RI, and CI of surplus food blind boxes purchase

#### Demographic characteristics of the respondents

The demographic variables of the subjects in the valid questionnaires are presented in Table 4.

**Table 4:** Demographic characteristics of the respondents

Sample	Category	Number	Percentage (%)
Gender	Male	252	44.29
	Female	317	55.71
Age	Under 18	5	0.88
	19–29	258	45.34
	30–39	257	45.17
	40–49	36	6.33
	Over 50	13	2.29
Marital status	Married	164	28.82
	Unmarried	405	71.18
Monthly Income (RMB)	Below 4000	75	13.18
	4001–6000	119	20.91
	6001–12,000	267	46.92
	12,001–18,000	72	12.65
	18,001 or more	36	6.33
Education	Junior high school or below	2	0.35
	High school or secondary school	17	2.99
	Undergraduate or college	502	88.23
	Institute including above	48	8.44
Occupation	Civil servant	46	8.08
	Clerk	276	48.51
	Worker	96	16.87
	Public service agency	40	7.03
	Student	53	9.32
	Self-employed	58	10.19
Area	Eastern china	345	60.63
	Central china	112	19.68
	Western china	78	13.71
	Northeast china	33	5.80
	Hong Kong, Macao and Taiwan in China	1	0.18

#### Measurement scale

Table 5 shows the questionnaire used in this stage of the survey, along with reference sources for variable codes, items, and scales.

**Table 5:** Measurement scale

Construct	Coding	Item	Source
PI	PI1	I would like to purchase surplus food blind boxes if they are available.	[56]
	PI2	If available, I will purchase surplus food blind boxes.	
	PI3	I plan to purchase surplus food blind boxes if they are available for purchase.	
	PI4	It would be my preference to purchase surplus food blind boxes if they are available.	
CI	CI1	It is my intention to continue purchasing surplus food blind boxes in the future.	[57]
	CI2	If given the opportunity, I would continue to buy food through surplus food blind boxes.	
	CI3	It is my intention to purchase surplus food blind boxes consistently throughout my daily life.	
	CI4	As long as surplus food blind boxes are available in the future, I am willing to continue to purchase them.	
RI	RI1	The surplus food blind boxes are something I would recommend to others.	[50, 58]
	RI2	It will be my pleasure to pass on the positive comments regarding the surplus food blind boxes to others.	
	RI3	In the future, I will recommend surplus food blind boxes to friends and relatives.	
	RI4	I will discuss the experience of surplus food blind boxes with my family and friends. (Removed)	
	RI5	I will brag about my attempts at surplus food blind boxes to others. (Removed)	

#### Reliability analysis

This study utilized SPSS to analyze the reliability of the data collected. The Cronbach’s Alpha and the corrected item-total correlation (CITC) were used as measures of reliability. Using Cronbach’s alpha, we can quantify the amount of random measurement error present in the overall score or average produced by a multi-item scale [60]. CITC is a measure of how closely one item in a test correlates with the items in other parts of the scale [61]. After the deletion of RI4 and RI5, the overall reliability of the questionnaire reaches the standard. Therefore, after the deletion of the above items, Cronbach’s Alpha was used to test the reliability of the remaining items. The reliability analysis results were presented in Table 6. Based on Table 6, the CITC for each constructs exceeds 0.4, indicating reliability of the items [62]. Currently, no matter which of the items are deleted, the new reliability value formed by the deletion is less than the current reliability value, which indicates that deleting items won’t be able to increase the reliability of the construct at this time. Additionally, currently Cronbach’s alpha values for each construct is greater than 0.6, indicating a relatively high degree of consistency between questionnaire and scale. This data can then be used in further analyses [63].

**Table 6:** Results of the reliability analysis

Construct	Item	CITC	Cronbach’s alpha if item deleted	Cronbach’s alpha
PI	PI1	0.714	0.828	0.865
	PI2	0.748	0.814	
	PI3	0.721	0.825	
	PI4	0.678	0.842	
RI	RI1	0.685	0.734	0.818
	RI2	0.646	0.774	
	RI3	0.681	0.740	
CI	CI1	0.750	0.839	0.878
	CI2	0.724	0.850	
	CI3	0.709	0.856	
	CI4	0.771	0.831	

#### Confirmatory factor analysis

This study used AMOS to conduct confirmatory factor analysis (CFA) on the data. A linear relationship exists between the latent variables, enabling subsequent analysis. A common latent factor method (CCLFM) was also calculated in this study to test for the presence of common method bias. It can be seen from the model fitting index Table 7 of CFA and CCLFM that the fit index of confirmatory factor analysis was satisfactory [64]. There was no significant difference between CCLFM and CFA in terms of model fitting results. The goodness-of-fit index (GFI), normed Fit Index (NFI), and comparative fit index (CFI) have the same values, root-mean-square error of approximation (RMSEA) and standardized root mean square residual (SRMR) have increased by 0.01, and adjusted goodness-of-fit index (AGFI) has decreased by 0.01, indicating that there was no common method bias problem in this study [65].

**Table 7:** Model fitting index comparison results of CFA and CCLFM

Common indices	χ2/df	RMSEA	GFI	AGFI	NFI	CFI	SRMR
Judgment criteria	< 3	< 0.06	> 0.95	> 0.95	> 0.95	> 0.95	< 0.06
CFA Value	1.588	0.032	0.980	0.967	0.981	0.993	0.020
CCLFM Value	1.628	0.033	0.980	0.966	0.981	0.993	0.020

In Table 8, the factor loading of all items was greater than 0.6, and the t-value was significantly greater than 1.96, indicating that no items need to be deleted. The squared multiple correlation (SMC) was greater than 0.5 [66]. A composite reliability (CR) of each construct was greater than 0.8, and the average variance extracted (AVE) was greater than 0.36 [67], indicating that each construct has good convergent validity.

**Table 8:** Results of the convergent validity test

Construct	Items	Factor loading	t-value	Standard error	*p*	SMC	AVE	CR
PI	PI1	0.784	21.411	0.157	0.001*	0.615	0.618	0.866
	PI2	0.822	22.959	0.172	0.001*	0.676		
	PI3	0.803	22.168	0.164	0.001*	0.645		
	PI4	0.731	19.402	0.194	0.001*	0.535		
RI	RI1	0.785	20.707	0.153	0.001*	0.616	0.601	0.819
	RI2	0.750	19.470	0.166	0.001*	0.562		
	RI3	0.790	20.894	0.154	0.001*	0.625		
CI	CI1	0.822	23.049	0.146	0.001*	0.676	0.646	0.879
	CI2	0.793	21.855	0.162	0.001*	0.629		
	CI3	0.765	20.733	0.158	0.001*	0.585		
	CI4	0.832	23.457	0.166	0.001*	0.692		

* The level of significance is 0.05

Data discriminant validity analysis was conducted simultaneously using the Fornell-Larcker criterion and Heterotrait-Monotrait ratio (HTMT). The discriminant validity analysis results were presented in Table 9. According to the results, the square root of the AVE of each construct exceeded the correlation coefficient of any other construct, reaching the suggested index of Fornell-Larcker [68]. Additionally, the HTMT calculation results for each construct were less than 0.8, reaching the recommended value [69]. It is evident that the constructs were well differentiated. Thus, all aspects of the study have a good discriminant validity.

**Table 9:** Results of discriminant validity

		PI	RI	CI
PI	Fornell-Larcker criterion	**0.786**		
	Heterotrait-Monotrait ratio	/		
RI	Fornell-Larcker criterion	0.615*	**0.775**	
	Heterotrait-Monotrait ratio	0.730	/	
CI	Fornell-Larcker criterion	0.657*	0.573*	**0.804**
	Heterotrait-Monotrait ratio	0.752	0.676	/

* Correlation coefficients are significant at 0.05

Note: The bold number is the square root of AVE

#### Structural equation model

**Fig. 3:**
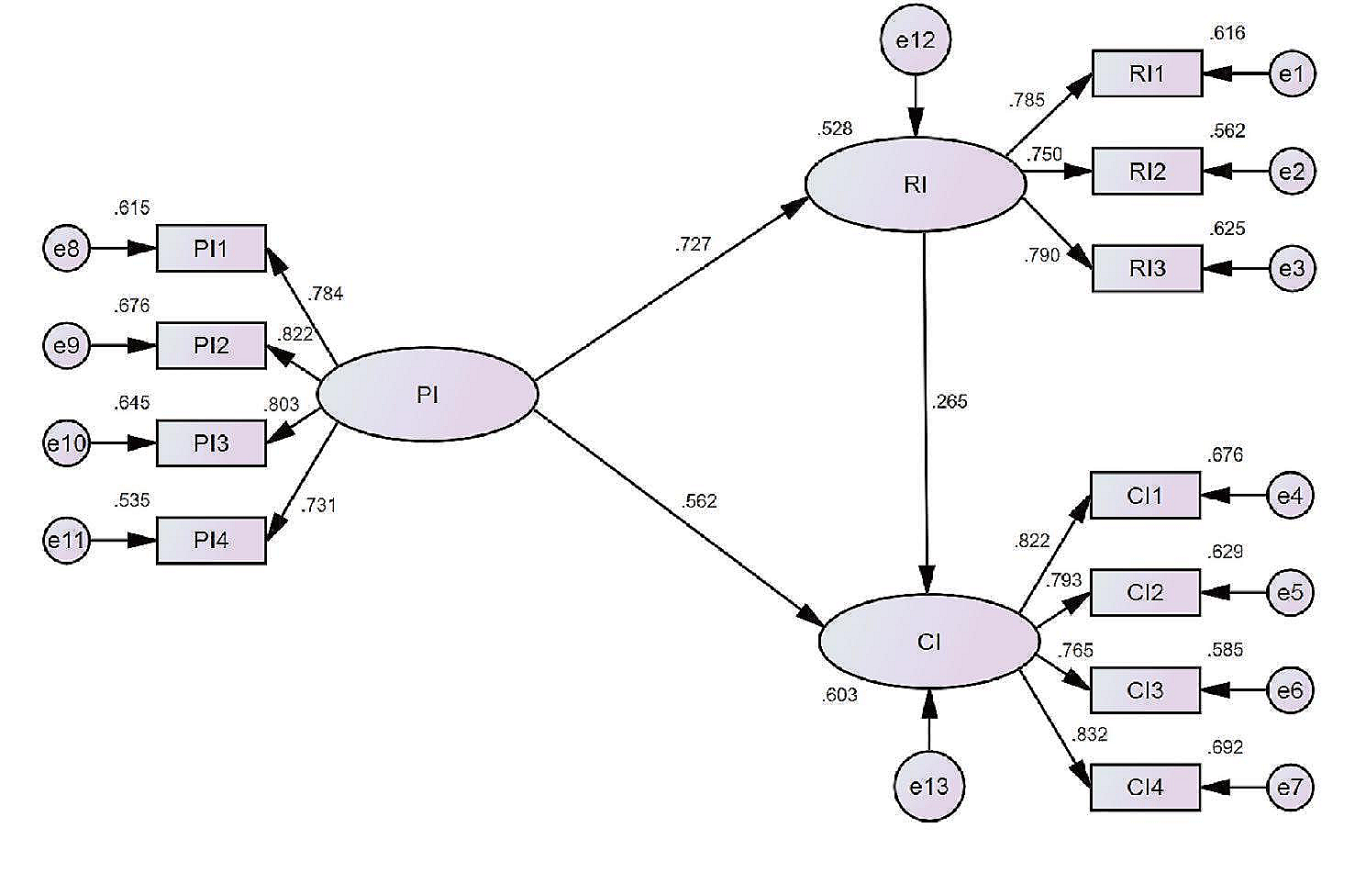
Results of the structural equation model framework

A structural equation analysis was used to model the data in this study, being presented in Fig. 3. It appears that all the paths in the model achieved the significant standard, indicating that there is a positive correlation between the constructs identified in the model. With a 95% confidence interval, we performed 2000 Bootstrapping calculations [70]. In Table 10, the fitting indicators of the model were higher than the recommended standards, indicating that the model was well fitted [64, 71, 72].

**Table 10:** Structural equation model fit

Common indices	χ2/df	RMSEA	GFI	AGFI	NFI	CFI	SRMR
Judgment criteria	< 3	< 0.06	> 0.95	> 0.95	> 0.95	> 0.95	< 0.06
Value	1.588	0.032	0.980	0.967	0.981	0.993	0.020

In Table 11, results of direct and indirect effects between constructs were presented, the direct influence path coefficient between PI and RI is 0.727, reaching the significant level (*p* <.05). Thus, H2 was valid. The user’s PI for surplus food blind boxes has a positive impact on the user’s RI. There was also a positive effect of RI on CI as a mediation variable. PI and RI have direct impact paths of 0.562 and 0.265 on CI, both reaching significant levels(*p* <.05). Thus, H1 and H3 was valid. Consumers were more likely to purchase when their recommendations were positive [73].

**Table 11:** Paths affect results

Path	Direct effect		Indirect effect		Total effect	
	β	Sig.	β	Sig.	β	Sig.
PI→RI	0.727	0.001*	/	/	0.727	0.001*
PI→CI	0.562	0.001*	0.193	0.001*	0.755	0.001*
RI→CI	0.265	0.001*	/	/	0.265	0.001*

* The level of significance is 0.05

According to Table 12, in this study, gender was evaluated as a moderator, and we used Chi-square minimum (CMIN) to test the moderation effect of gender on each influencing path. In this study, gender did not have a moderating effect. Food-related research has demonstrated that gender has a moderating effect on food interest and food quality [74, 75]. However, this study did not find that gender has a moderating effect between PI, RI, and CI of surplus food blind boxes.

**Table 12:** Results of mediation effect

Moderating variable	Independent variable	→	Dependent variable	CMIN	*p*
gender	PI	→	RI	0.249	0.618
	PI	→	CI	2.290	0.130
	RI	→	CI	2.797	0.094

* The level of significance is 0.05

## Discussion

The research consisted of 2 studies, which examined how to increase consumers’ PI of surplus food blind boxes. Study 1 is a study from the perspective of the users, collecting and analyzing the factors that influence the purchase of surplus food blind boxes by users. In study 2, we use a structural equation model to examine the relationship between the PI, RI and CI. In this research, some important findings are presented regarding the marketing of surplus food blind boxes, which are discussed in more detail below.

Based on the results of Study 1, surplus food blind boxes may be designed in a manner that strengthens factors that are likely to encourage consumer purchases. No matter what marketing methods food merchants employ, ensuring the quality of their products is paramount. Blind boxes containing surplus food are intended to provide food, not to serve as decorations. Therefore, food merchants must not only pay attention to the external packaging design and marketing methods of blind boxes, but also to the quality of the products contained therein. The merchant can promote the concept of “fresh leftovers” in the surplus food blind boxes during the marketing process, emphasizing the food safety and quality assurance of the boxes. Meanwhile, if merchants are capable of consistently ensuring good food quality, so that consumers have a high level of trust in the brand, this may also encourage them to purchase new products such as the same brand’s surplus food blind boxes [76]. Secondly, as the ecological environment continues to deteriorate around the world, overpopulation and resource shortages are forcing humans to rethink their relationship with nature. A surplus food blind box is an environmentally friendly green product that reuses leftover edible food. Some consumers with an awareness of the environment make green purchases as a result of surplus food blind boxes’ eco-friendliness [77]. There is promising potential for promoting sustainable development through eco-innovation and ecological consumption [78]. Studies have shown that consumers are more likely to accept or purchase environmentally friendly products out of a sense of environmental responsibility [79]. Therefore, we suggest that we might consider increasing PI by using eco-friendly elements on the surplus food blind boxes purchases, such as the use of expressions like “eco-friendly” and “environmentally safe” in packaging design. It is important to increase consumers’ environmental awareness and sense of responsibility for nature and society, and to inspire green consumption behavior with surplus food blind boxes. Lastly, it must be noted that, whilst some consumers are motivated by environmental concerns to choose surplus food blind boxes, it cannot be ignored that there are some consumers who are motivated by conspicuous consumption rather than moral concerns. A surplus food blind box has the moral value of being green and environmentally friendly, while at the same time being in line with the current fashion trend for blind boxes. Social networking has become a huge influencer in the new media era, as users are more easily influenced with information in social media. At the same time, users will actively display or demonstrate their experiences using products on social networks, thereby influencing others [25]. It is possible for merchants to take advantage of the conspicuous psychology of these consumers to market surplus food blind boxes, create a new fashion trend of surplus food blind boxes, and guide consumers in the direction of the purchase.

Furthermore, during the marketing design process of surplus food blind boxes, consider weakening or avoiding the factors that negatively impact consumer behavior. Firstly, the results of this study indicate that consumers’ perceptions of food risks are associated with surplus food blind boxes. A blind box shopping experience can be compared to an adventure, and the suspense of the blind box can evoke a sense of anticipation in some consumers. Consumers’ purchase decisions are affected by whether the surplus food blind boxes contain fresh food and whether they will cause adverse health effects after consumption. For sustainable clothing sales, it is necessary to make the product production process transparent in order to alleviate users’ doubts about the merchant’s propaganda [40]. The marketing process of surplus food blind boxes may offer opportunities to learn about making the production process transparent, so that users can track the process of food manufacture, packaging, and transportation. This can reduce users’ concerns about food risks and increase the surprise feeling brought by the blind box. Second, some consumers object to the method of selling surplus food in blind boxes. As we discovered, some consumers are concerned that they will be looked down if they purchase surplus food blind boxes; others are concerned that the food in the boxes will be consumed by others, which is psychologically unacceptable; and others dislike the packaging of blind boxes, finding the purchase process tedious and cumbersome. The following solutions can be adopted for different reasons of resistance. Firstly, through social media and public figures, etc., rebuild consumers’ understanding of the consumption of surplus food. Let consumers rethink a series of environmental pollution caused by the waste of surplus edible food. Consumer perceptions of food risks have been shown to directly influence their purchasing decisions in the catering industry [80]. Thus, we might speculate that dispelling the belief that food blind boxes are leftovers from others may increase consumers’ willingness to try them by reducing perceived risks. A shift in stereotypes is beneficial not only for the sustainable marketing of surplus food blind boxes, but also because consumers will be emotionally aware of the importance of protecting the environment, allowing them to take measures to avoid food waste in other areas of their daily lives as well. Secondly, for individuals who are dissatisfied with the outer packaging design of blind boxes and find it boring, merchants can develop optimized designs and adjustments based on their own product characteristics, combined with the characteristics of the main consumer groups, and use surplus food blind boxes to develop special activities, such as themed marketing related to a festival or a specific period. Thirdly, consumers were concerned about the taste of surplus food blind boxes, which has a negative impact on their PI. Although blind box sales are marketed as a surprise strategy, there are still risks involved [40]. Those consumers who are interested in taking risks may experience excitement, while those who are risk averse may suffer psychological damage because of the products in the blind box not living up to their expectations [10, 81]. Since consumers have different risk cognitions, refining the classification of blind boxes can reduce users’ anxiety as a result of uncertainty. For example, merchants could provide completely random blind boxes for adventurous customers, and at the same time divide the blind boxes by taste so that consumers who like sweets could choose from this area only. This would reduce users’ dislikes of the food. Moreover, merchants can provide consumers with a more direct taste experience through tasting. Fourth, the marketing design of surplus food blind boxes is inadequate. Currently, surplus food blind boxes are sold exclusively through limited channels, as surplus foods are difficult to determine the type of daily food, and sometimes they may be relatively single in composition. Studies have shown that food is one of the most popular products in online shopping, with a growth rate of 12% per year [82]. Clearly, the food market has significant consumption potential. In the surplus food blind boxes marketing model, users are usually required to go to the store to pick up the blind boxes after placing an online order. When ordered online, takeout food is more convenient than blind boxes of leftovers that must be picked up in a store. However, going into a physical store can enable consumers to see more real products and therefore understand the product more intuitively. By interfacing with sales staff, a consumer may be able to gain greater trust in the brand, hence increasing their likelihood of making a purchase [83–85]. Public welfare concepts can be promoted through surplus food blind boxes. For instance, by purchasing surplus food blind boxes, contacting members of the community, and disseminating the environmental protection concept of surplus food blind boxes. The literature indicates that brands that promote eco-friendly lifestyles and environmental protection, i.e., brands with environmental corporate social responsibility, have a positive impact on user awareness of the environment [86, 87]. It is important to note that food blind boxes seem to be distinct from greenwashing behavior. In other words, consumers who support environmental protection may have a positive view of the brand, thereby promoting the company’s image. Customers will be more likely to become brand advocates if they have a positive impression with the blind box. Active customer groups are more willing to recommend a product to others if they have a positive experience with the blind box [40]. Based on our study results, RI is also shown to lead to CI, which is positive for the sustainable marketing of surplus food blind boxes. In today’s world, merchants cannot ignore the benefits that social media and customer recommendations provide. Additionally, the surplus food blind boxes can be customized for different groups of people to enhance the user’s experience and increase their PI.

We established the path relationship between PI, CI, and RI in study 2. The study shows that it is possible to strengthen the RI for surplus food blind boxes to promote consumers’ CI of surplus food blind boxes during the marketing process. According to the results of structural equation modeling, the hypotheses presented in this paper are confirmed. Accordingly, H1 is valid, indicating a significant positive correlation between PI and CI for users purchasing surplus food blind boxes. It may be that the consumer decides to purchase surplus food blind boxes for the first time out of curiosity or from their concern for the environment. If they are able to improve their experience, they may be more likely to purchase them in the future. Results of this study confirm previous studies that have shown a positive relationship between PI and CI [88]. In addition, it may also be influenced by a number of factors, including personal characteristics, situational factors, and product-related factors [88]. Marketers should understand and combine these factors in order to adjust marketing strategies to help users increase their PI and CI. The hypothesis H2 is valid, indicating that the PI of users who purchase blind boxes of surplus food has a significant positive correlation with the RI. Following the purchase and actual experience of the product, users will provide feedback regarding their experience with the product. There is no doubt that offering a better purchasing experience to the customer will help to build a good reputation for the surplus food blind boxes. In today’s competitive consumer market, although users have many choices, they also add many obstacles [89]. This type of marketing method can improve the user experience and provide users with the opportunity to make quick decisions. A stronger quality of food in surplus food blind boxes, enhancement of the types of products inside surplus food blind boxes, and improvement of the quality of the surplus food blind boxes may attract more consumers and result in positive recommendations. The hypothesis H3 is valid, indicating that the RI of users buying surplus food blind boxes correlates significantly with CI. It is possible for consumers to gain more information about surplus food blind boxes by seeking recommendations from others. The information provided by consumers who have no vested interests and are more independent is more convincing and credible than the information provided by marketers [90]. A good reputation can reduce user uncertainty, improve users’ trust in the product of surplus food blind boxes, and aid in continuous purchase decisions. According to a study on gourmet tourism tourists, the more people recommend dishes, the more sensory appealing they are, and the more likely consumers are to consume them and return to them [91]. Research on system recommendations and user intention to pay for online retail has shown that personalized recommendations will positively influence the purchase decision of users [92]. Researchers have found that consumers’ RI has a positive impact on CI when it comes to surplus food blind boxes.

## Conclusions

### Theoretical contributions

The study examines the reasons for the positive and negative impacts on consumers’ PI of surplus food blind boxes and establishes a theoretical framework linking surplus food blind boxes PI, RI, and CI. It is intended to fill the gap in research on how to increase the intention of consumers to purchase surplus food blind boxes. This study examines the reasons consumers buy surplus food blind boxes and analyzes the causes and suggests possible solutions to these issues. While gender did not have a moderating effect on the surplus food blind boxes in this study, it is possible that gender might have a moderating effect in certain types of blind box purchases.

Additionally, this study develops a theoretical framework between PI, RI and CI and found that PI has a positive influence on RI and CI, with RI serving as a mediator between PI and CI. This model is not limited to the marketing of surplus food blind boxes but can also be used to promote other new consumption models, and can serve as a reference for surplus food blind box merchants and other practitioners.

### Practical contributions

These recommendations are provided for reference in the actual marketing management of surplus food blind box merchants as a result of the research conducted in this paper. To begin with, it is important to strengthen the factors that encourage consumers to purchase surplus food blind boxes.

1. Ensure food quality. Pay careful attention to the quality of the food in the surplus food blind box and make sure that the product has a positive reputation.
2. Enhance sustainability perception. Using surplus food blind boxes as a marketing tool to enhance consumer awareness of sustainability and inspire them to develop sustainable consumption habits may be an effective solution.
3. Enhance public understanding. It is important to inform conspicuous consumers of the importance of surplus food blind boxes through social media and other publicity methods.

Additionally, improve the negative factors that influence consumers’ decision to purchase surplus food blind boxes.

1. Transparent production process. With transparent surplus food blind box production and packaging, users can monitor the manufacturing process of the product, increase user awareness of the product, and encourage user consumption of the product.
2. Develop marketing strategy. It is important to improve the marketing strategy according to the specific reasons why the main user groups of the brands are reluctant to buy surplus food blind box products.
3. Classification by groups. Classifying surplus food blind boxes based on the characteristics of different types of food consumption groups.

In conclusion, RI can serve as an effective tool to link the relationship between PI and CI. Using an online and offline marketing strategy, recommend surplus food blind box products and promote them through online and offline platforms. The use of social media platforms, celebrity endorsements, etc. will allow the brand to be communicated and brand loyalty increased by using those methods.

### Limitations and future research

There are still several limitations that can be addressed through future research. First, the sample of this study is limited to China, but future research can examine consumer groups in other regions of the world. Second, further research and analysis can be conducted on specific consumer groups of different types of food merchants in future studies, as well as examining consumers of different genders. Third, in this study, we examine the value of surplus food blind boxes from the perspective of food waste. However, future research can examine it from multiple perspectives such as the social value, which is an important part of promoting sustainable marketing. Fourth, the structural equation model based on study 2 may be expanded to include more research factors in the future for a more systematic and comprehensive analysis.

Last, this study used an online survey method which may lead to less focus among the subjects than an offline survey. Further, our sampling method is limited by objective factors such as manpower and funds, and it only represents a part of the audience that we hope to survey. In the future, face-to-face in-depth surveys can be conducted with a large number of subjects from a variety of cultural backgrounds on a conditional basis.

### Electronic supplementary material

Below is the link to the electronic supplementary material.

Supplementary Material 1

## Data Availability

The data that support the findings of this study are available from the corresponding author upon reasonable request.
