# Supplementary material for: Is there any way to increase consumers’ purchase intention regarding surplus food blind-boxes? An exploratory study
Source: BMC Psychol. 2024 Feb 28;12:103. doi: 10.1186/s40359-024-01587-y (PMC10900719; doi:10.1186/s40359-024-01587-y)
Supplement: Supplementary file 1 — Supplementary Material 1 [file 40359_2024_1587_MOESM1_ESM.docx]

**Appendix**

**The first round of questionnaires ( two open-ended questions)**

1.What are three reasons positively influencing your purchase intention towards surplus food blind-boxes?

2. What are three reasons negatively influencing your purchase intention towards surplus food blind-boxes?

**The second round of questionnaires (50 new factors)**

*Positive*

1. The cost effectiveness of surplus food blind-boxes is what makes me more inclined to purchase surplus food blind-boxes.

2. I find surplus food blind-boxes to be novel and interesting, which increases my intention to purchase it.

3. The use of surplus food blind-boxes can reduce food waste in this way, which is why I am more inclined to purchase it.

4. Blind boxes appeal to me because they provide a sense of surprise, which is the reason why I am intent to purchase surplus food blind boxes.

5. My intention to purchase surplus food blind-boxes is strengthened by the convenience and speed of the purchase process.

6. My intention to purchase surplus food blind-boxes is fueled by my curiosity to try this form.

7. Surplus food blind-boxes are an environmentally friendly method, which is why I will be more inclined to buy it.

8. Due to the availability of various types of food, I am more willing to purchase surplus food blind-boxes.

9. It is the taste of the food that motivates me to purchase surplus food blind-boxes.

10. Having the opportunity to taste new foods is what makes me more inclined to buy surplus food blind-boxes.

11. A feeling of necessity and practicality in life is what encourages me to purchase surplus food blind-boxes.

12. My intention to purchase surplus food blind-boxes is fueled by the opportunity to pick up cheap and try your luck.

13. The large amount of food makes me more willing to purchase surplus food blind-boxes.

14. My intention to purchase surplus food blind-boxes is strengthened by the feeling that it is a fashion trend.

15. This was an educational experience, which strengthened my intention to purchase surplus food blind-boxes.

16. By purchasing surplus food blind-boxes, resources can be fully utilized, which strengthened my intention to purchase surplus food blind-boxes.

17. Blind box packaging design is appealing to me, which strengthened my intention to purchase surplus food blind-boxes.

18. Blind box food is of high quality, which strengthened my intention to purchase surplus food blind-boxes.

19. This novel combination of ingredients in the blind box strengthens my intention to purchase surplus food blind boxes.

20. The recommendation of surplus food blind-boxes by others strengthens my intention to purchase it.

21. The sense of superiority and accomplishment I gain from purchasing surplus food blind-boxes strengthens my intention to purchase it.

22. It is the guarantee of food hygiene that strengthens my intention to purchase surplus food blind-boxes.

23. Food in blind-boxes can be stored, which strengthens my intention to buy surplus food blind-boxes.

24. Food safety is guaranteed in blind boxes, which strengthens my intention to buy surplus food blind-boxes.

25. Reasonable nutritional combination of the blind boxes, which strengthens my intention to buy surplus food blind-boxes.

*Negative*

1. The fear that the surplus food blind-boxes may be expired and not fresh as advertised reduces my intention to buy surplus food blind-boxes if they are expired.
2. The concern I have about the quality of surplus food has reduced my intention to buy surplus food blind-boxes because I am concerned about the quality.
3. As a result of worrying about buying a food I don't like, I am less willing to purchase surplus food blind-boxes.
4. My intention to purchase surplus food blind-boxes is reduced by concerns about the hygienic condition.
5. I am less inclined to buy surplus food blind-boxes because of the lack of flavor.
6. Due to the fear of being cheated, the intention to purchase surplus food blind-boxes has decreased.
7. Surplus food blind-boxes are too expensive, which reduces my intention to purchase it.
8. Due to my concerns about food safety, I have reduced my intention to purchase surplus food blind-boxes.
9. Due to the uncertainty of the unknown, I am less inclined to purchase surplus food blind-boxes.
10. My do not intent to purchase surplus food blind-boxes is due to my psychological aversion to surplus food.
11. My concern about the health effects of surplus food blind-boxes leads me to reduce my intention to purchase it.
12. Due to the format of surplus food blind-boxes, I am less willing to purchase it.
13. I am less likely to purchase surplus food blind-boxed because there is no demand for it.
14. Due to the idea that leftover food has been eaten by others and is unhealthy, I am less likely to buy surplus food blind-boxes in the future.
15. In my opinion, buying surplus food blind-boxes is boring, which is why I have a reduced intention to purchase it.
16. Due to my fear of losing face in front of others, I have reduced my intention to purchase surplus food blind boxes.
17. As a result of the timing of purchases and the limited channels, I am less likely to purchase surplus food blind-boxes.
18. I am less likely to purchase surplus food blind boxes due to the troublesome and inconvenient purchase method.
19. Due to my concern about not having enough food within the blind-box, I am less willing to purchase surplus food blind-boxes.
20. My do not intent to purchase surplus food blind boxes decreases if I encounter food that I do not like.
21. Due to concerns that the food in the blind box is too single, I am less likely to purchase surplus food blind boxes.
22. Due to my concern about the psychological gap and disappointment, I am less inclined to purchase surplus food blind-boxes.
23. Having a limited storage time for surplus food reduces my intention to buy surplus food blind-boxes.
24. The appearance of surplus food blind-boxes is not my favorite, which reduces my intention to purchase it.
25. Lack of relevant management system constraints is what reduces my intention to purchase surplus food blind-boxes.
